# Rare Multiple Brain Metastases Following Debulking Surgery and Androgen Deprivation Therapy in Aggressive Prostate Cancer-Case Report

**DOI:** 10.32604/or.2025.066478

**Published:** 2026-01-19

**Authors:** Andong Cheng, Yiding Chen, Hao Li, Feixiang Yang, Junlan Jiang, Sheng Tai, Weiwei Chen, Yu Guan, Shuiping Yin, Jialin Meng

**Affiliations:** 1 Department of Urology, The First Affiliated Hospital of Anhui Medical University, Institute of Urology, and Anhui Province Key Laboratory of Urological and Andrological Diseases Research and Medical Transformation, Anhui Medical University, Hefei, 230001, China; 2 School of Life Sciences, Anhui Medical University, Hefei, 230032, China; 3 Department of Pathology, The First Affiliated Hospital of Anhui Medical University, Hefei, 230001, China; 4 Department of Neurosurgery, The First Affiliated Hospital of Anhui Medical University, Hefei, 230001, China

**Keywords:** Prostate cancer, brain metastases, metastatic prostate cancer, castration-resistant prostate cancer, case report

## Abstract

**Background:**

In clinical practice, approximately 80% of prostate cancer (PC) cases are localized and can achieve favorable outcomes with appropriate treatment. Conversely, some remaining cases exhibit an aggressive phenotype or develop resistance to therapeutic interventions, leading to tumor metastasis and a poorer prognosis. When PC metastasizes to distant sites, the bone remains the predominant location, and brain metastases are regarded as exceedingly rare.

**Case Description:**

The current study focused on a rare clinical PC case that presented multiple brain metastases after prostate surgery. The patient was initially diagnosed with PC through prostate biopsy and subsequently underwent prostate debulking surgery while continuing androgen deprivation therapy, which maintained low prostate-specific antigen (PSA) levels for 4 years. However, a sudden PSA surge to 7.858 ng/mL led to the emergence of two brain metastatic tumors, which were confirmed to have originated from the prostate.

**Conclusions:**

Patients with advanced PC require comprehensive evaluations to detect rare metastatic sites, such as the brain, to avoid missed diagnoses. For patients with brain metastases, a multimodal approach combining surgical resection, postoperative radiotherapy, and endocrine therapy can effectively alleviate symptoms and enhance survival.

## 1 Introduction

Prostate cancer (PC) is the second most common malignancy in men worldwide. Approximately 80% of cases are localized to the prostate and can typically be effectively managed with surgery or endocrine therapy [1–3]. However, some cases may develop into castration-resistant PC (CRPC) or present distant metastases during treatment [4]. Distant metastases occur in most cases of advanced PC and are the leading cause of mortality in patients with PC [5].

Bone is the predominant site of distant metastasis in PC. Approximately 65%–75% of patients with advanced PC are diagnosed with bone metastases at initial diagnosis [6,7]. Hematogenous dissemination to the brain and central nervous system is uncommon [8,9]. PC cell invasion disrupts the osteoblastic-osteolytic balance, resulting in abnormal bone formation [10]. The brain microenvironment is a crucial determinant of brain metastasis progression. Brain-metastatic tumor cells can traverse the blood-brain barrier, evade immune surveillance, and adapt to the distinct conditions of the brain microenvironment [10]. Notably, lung cancer, breast cancer, and melanoma exhibit a high propensity for brain metastasis [11,12]. Studies have reported that the brain metastases from PC range from approximately 0.16% to 0.63% [13,14]. However, as the survival period of patients with PC extends, the brain metastases also increase. This relatively low incidence has led to limited relevant information. Research indicates that prostate brain metastasis may be associated with defects in homologous recombination repair and DNA methylation remodeling [15,16]. Furthermore, studies have revealed significant variations in PSA levels with brain metastases, and these levels may remain normal despite extensive metastatic disease [17,18].

Brain metastases are typically detected only after central nervous system symptoms or postmortems due to their occult nature and low incidence [8,19]. Treatment options for brain metastases from PC remain limited and have a poor prognosis. Therefore, early diagnosis and timely intervention are crucial. This study reports a clinical case of multiple brain metastases following cytoreductive surgery for PC, providing a valuable reference for clinical practice.

This study was approved by the clinical research ethics committee of the First Affiliated Hospital of Anhui Medical University ethics review approval, with the reference number: PJ 2025-05-17. The handwritten informed consent was obtained from the patient. This study was prepared according to the CARE case report guideline, and a CARE checklist was provided. Please see Supplementary Material S1 for more details.

## 2 Case Report

**Figure 1:**
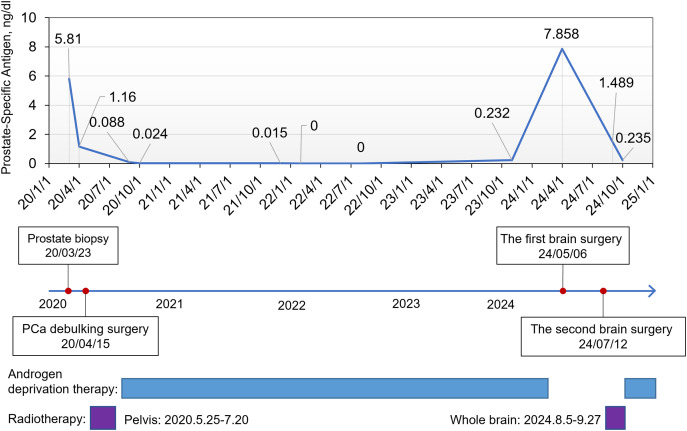
The overall treatment course of the patient and prostate-specific antigen (PSA) change curve

Herein, we present a rare case of PC with multiple brain metastases. The overall diagnostic and treatment processes are illustrated in Fig. 1.

**Figure 2:**
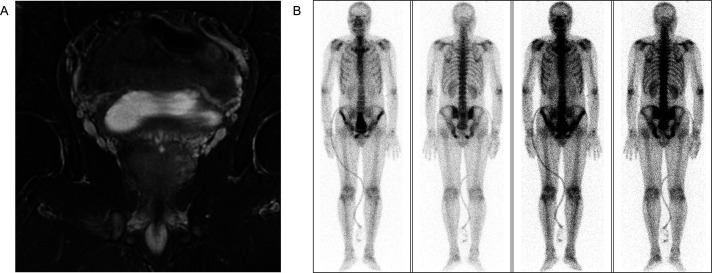
Preoperative imaging evaluation. (**A**) Prostate MRI. (**B**) Bone scintigraphy

**Figure 3:**
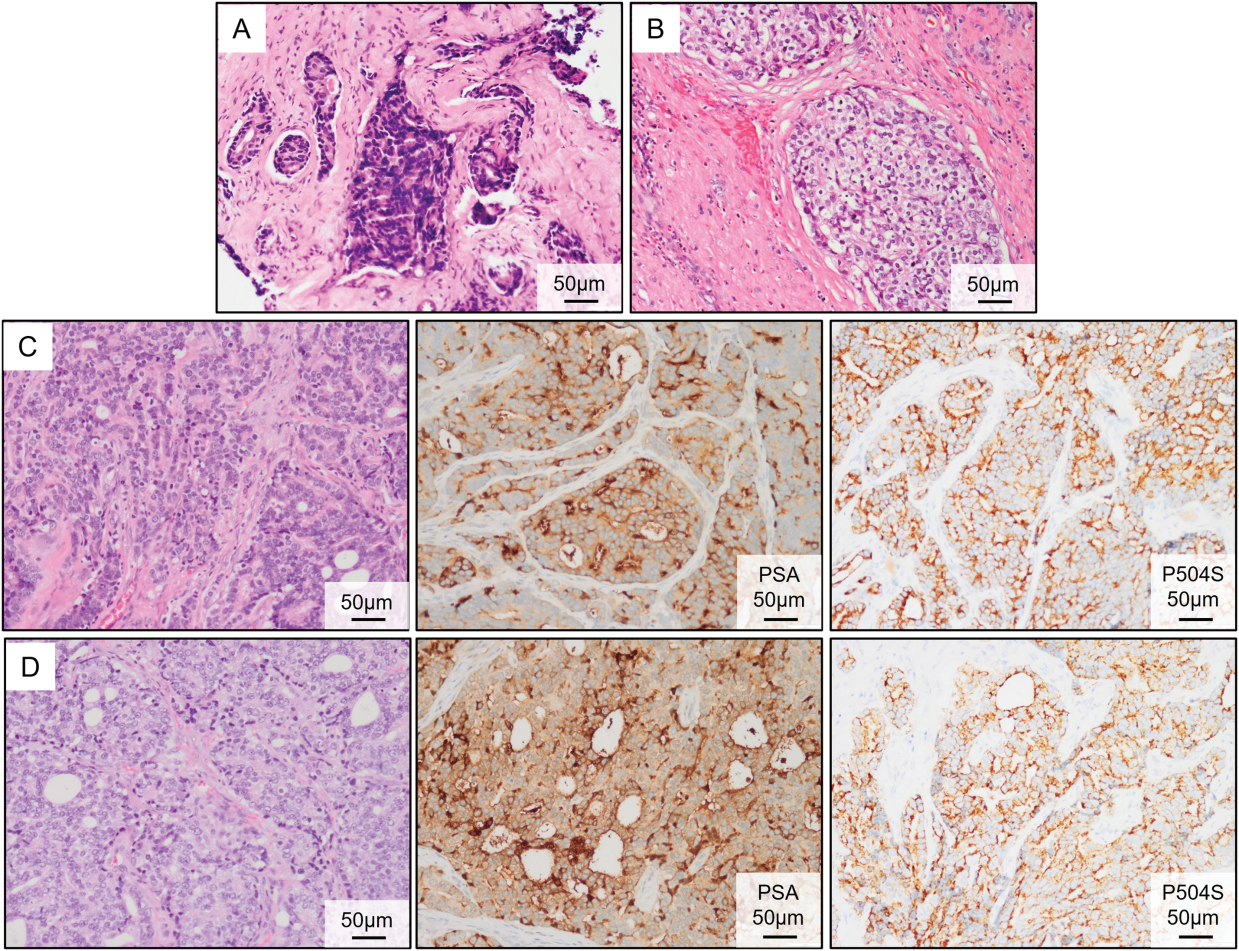
Pathological results of the primary PC and brain metastasis tumors. (**A**) Hematoxylin and eosin (HE) staining of the prostate biopsy tissue. (**B**) Postoperative HE staining of the PC tissue. (**C**) HE staining and immunohistochemical (IHC) (PSA and P504S) of the metastatic tumor following the first brain surgery. (**D**) HE staining and IHC (PSA and P504S) of the metastatic tumor following the second brain surgery

A 51-year-old man was admitted to the First Affiliated Hospital of Anhui Medical University in April 2020 with a six-month history of intermittent dysuria, accompanied by urinary frequency, urgency, and pain. The serum PSA level of the patient was 5.810 ng/mL at the outpatient visit, and urodynamic testing confirmed an overactive bladder. The prostate nuclear magnetic resonance imaging (MRI) revealed a prostate size of approximately 35.0 mm × 36.2 mm × 45.6 mm (Fig. 2A). A long T1 and short T2 signal was observed in the right peripheral and central zones, accompanied by a high diffusion-weighted imaging (DWI) signal and a significantly reduced apparent diffusion coefficient value. The bladder was moderately filled with a normal wall thickness. No significantly enlarged lymph nodes were detected in the pelvic or bilateral inguinal regions, and fluid accumulation was not observed in the pelvic cavity. Bone scintigraphy revealed increased metabolic activity in the bilateral ischial pelvis (Fig. 2B). Subsequently, a 12-core ultrasound-guided prostate biopsy was performed, and the pathological results confirmed that all 12 cores were prostate acinar cell carcinomas, while perineural invasion was observed in five cores. Gleason scores were as follows: One core 5 + 5 = 10, six cores 4 + 5 = 9, four cores 5 + 4 = 9, and one core 4 + 4 = 8 (Fig. 3A). After thorough discussions with the patient and family, a robot-assisted laparoscopic radical prostatectomy with pelvic lymph node dissection was scheduled. Intraoperatively, the prostate was firmly adherent to the rectal and right pelvic wall, rendering complete resection unfeasible. Consequently, a prostate debulking surgery was performed, with partial prostate tissue, bilateral vas deferens, and seminal vesicles resection, and bilateral pelvic lymphatic adipose tissue removal. Postoperative pathology report confirmed this case as prostatic adenocarcinoma, with an overall Gleason score of 4 + 5 = 9 (Fig. 3B). Cancerous tissue was observed in the right and left lobes and the prostatic apex. The tumor burden was approximately 90%. Perineural invasion was present (+). Tumor emboli presence in interstitial blood vessels cannot be excluded. No definitive tumor involvement was observed at the specimen margins. No cancerous tissue was detected in the seminal vesicles, and no definite metastasis was observed in the right and left pelvic lymph nodes. The patient was successfully discharged after surgery and subsequently received androgen deprivation therapy with bicalutamide. The patient also received 95% PTV1: 7000c Gy/35f/48d, 95% PIV2: 5000c Gw25f/36d, for a total of 35 pelvic radiotherapy sessions from May 25 to July 20. No abnormal PSA elevation was observed during the 3.5-year follow-up.

**Figure 4:**
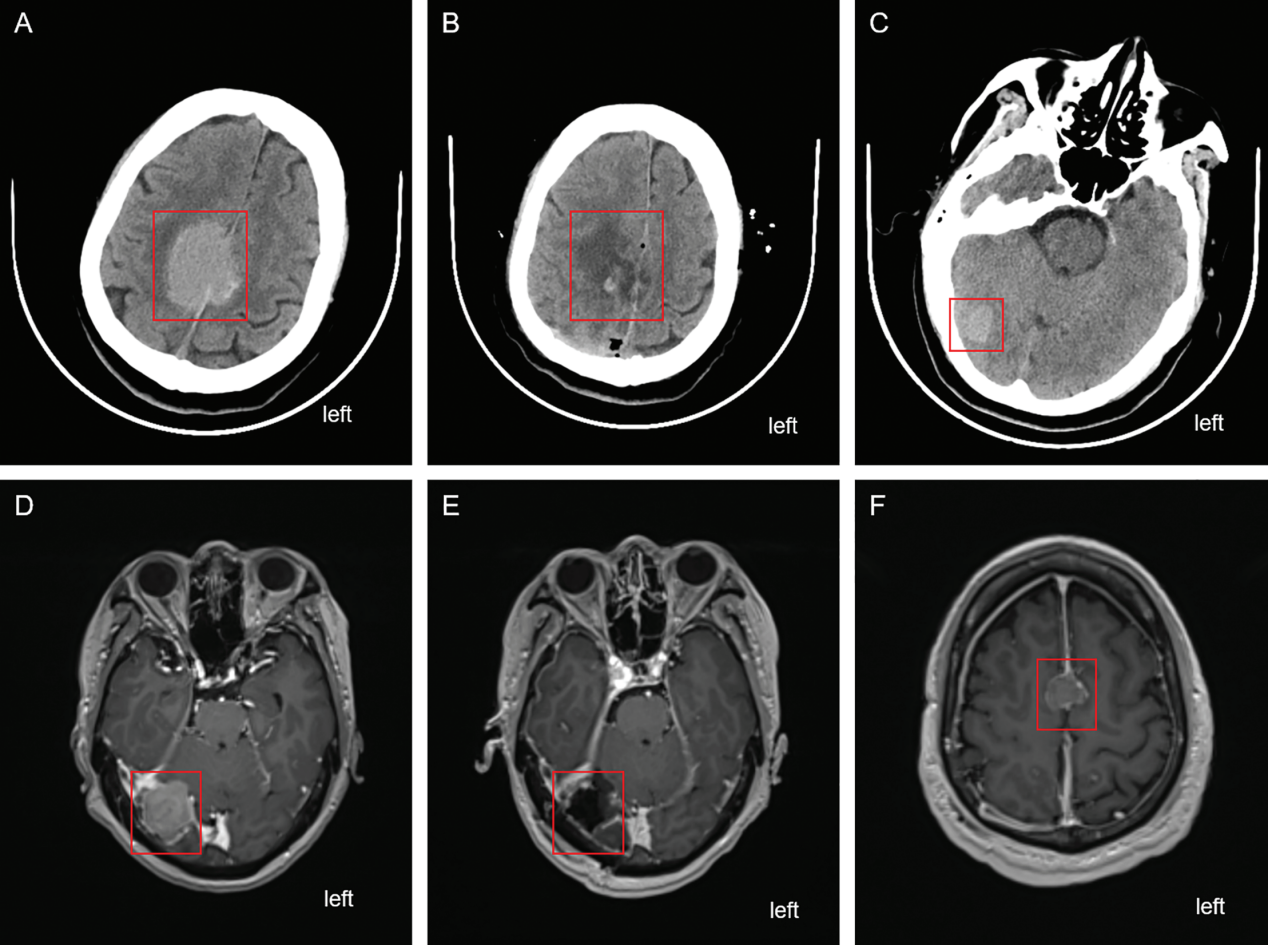
Cranial imaging examination before and after the two brain surgeries. (**A**) CT scan depicting the falx cerebri tumor before the first brain surgery. (**B**) CT depicting the falx cerebri region after the first brain surgery. (**C**) Unresected tumor in the right occipital region after the first brain surgery. (**D**) Enhanced MRI revealing the enlarged right occipital tumor before the second brain surgery. (**E**) Enhanced MRI depicting the right occipital region post the second brain surgery. (**F**) Recurrent and unresected tumor in the frontal lobe region. The red box highlights the location of the tumor lesion

On 16 April 2024, the patient presented to the oncology clinic with a two-week history of left lower limb weakness. At that time, the PSA level of the patient was 7.858 ng/mL. Brain computed tomography (CT) revealed a round high-density lesion in the falx cerebri and a patchy high-density lesion in the right occipital lobe, both with indistinct margins (Fig. 4A). Brain MRI, including plain, contrast-enhanced, and DWI, revealed abnormal lesions in the bilateral frontoparietal falx cerebri and right occipital region. These lesions appeared isointense on T1-weighted imaging and slightly hyperintense on T2-weighted imaging, measuring approximately 4.2 cm × 3.4 cm and 1.9 cm × 1.6 cm, respectively. The surrounding brain parenchyma was compressed. The lesion exhibited mildly hyperintense signals on DWI and significant enhancement after contrast administration, with tortuous vascular shadows visible at the lesion margins. The patient was admitted to the neurosurgery department of our hospital on April 25. Considering the medical history, imaging findings, and the poor overall condition of the patient, lumbar cistern drainage and microsurgical resection of the cerebral falx lesion were performed (Fig. 4B). Due to the small size of the right occipital mass, it was not resected during the initial surgery (Fig. 4C). The pathological report of immunohistochemistry analysis revealed α-methylacyl-CoA racemase (P504S; +), PSA (+), tumor protein 63 (P63; -), high-molecular-weight cytokeratin (34βE12; -), Ki67 (~30% +), cytokeratin 20 (CK20; -), cytokeratin 7 (CK7; -), confirming that the brain tumor originated from PC (Fig. 3C). The patient recovered well postoperatively and was discharged in stable condition. At the two-month outpatient follow-up, contrast-enhanced brain MRI revealed a right occipital lobe mass enlargement, measuring approximately 38 mm × 22 mm (Fig. 4D). After informing the patient and their family about the condition and obtaining consent, a right-sided arcuate craniotomy for lesion resection was performed (Fig. 4E). Postoperative immunohistochemistry analysis revealed PSA (+), P504S (+), CK20 (−), and CK7 (-), confirming that the right occipital mass originated from PC (Fig. 3D). A recurrent tumor was also detected at the initial surgical site in the falx cerebri (Fig. 4F). However, given the compromised health condition of the patient, alternative treatment strategies were considered, and surgery was not performed. The patient was discharged in stable condition after surgery and subsequently underwent 20 sessions of whole-brain radiotherapy (WBRT) between August 5 and September 27, 2024, along with ADT maintenance, and PSA levels were maintained at a low level (Fig. 1).

## 3 Discussion

PC is a significant global health burden and the third most prevalent malignancy in males, primarily affecting older individuals, with nearly 430,000 deaths reported annually [20]. The standard clinical treatment for PC includes surgical intervention and ADT. ADT can be achieved through surgical or pharmacological castration, leading to a significant reduction in testosterone levels. However, most patients undergoing ADT develop acquired therapeutic resistance within 24 months of treatment initiation, ultimately progressing to metastatic CRPC (mCRPC) [4,5]. Although recent studies have contributed to advancements in the diagnosis and clinical management of PC, treatment options for advanced or aggressive cases remain limited, resulting in a poor prognosis and reduced survival [2].

The clinical presentation of PC can progress from asymptomatic, low-risk localized disease to aggressive metastatic disease [20]. Approximately 17% of PCs develop metastases, with the bones being the most common site, followed by distant lymph nodes, liver, and lungs [2,3,21]. The incidence of brain metastases from PC is low, and subtle early symptoms often prevent routine clinical surveillance for brain involvement. The lack of surveillance has contributed to the increased occurrence and progression of brain metastases from PC. Brain metastases are typically detected in the advanced stages of PC, often presenting as multiple lesions [22–24].

PSA was first identified as a biomarker for PC in 1987, and studies have demonstrated that larger tumor volumes correlate with higher PSA levels [25]. Current guidelines advocate a risk-adapted screening strategy based primarily on baseline PSA levels. Men with PSA levels <1 ng/mL at age 40 or <2 ng/mL at age 60 are considered at low long-term risk for metastatic PCa or disease-specific mortality [26–28]. However, in this patient, the PSA level was only 5.810 ng/mL at first diagnosis, yet the prostate biopsy Gleason score was high, 5 + 5 = 10, and prostate adherent to rectal and pelvic walls were observed in the prostate surgery. During the two years of ADT and follow-up, the PSA of the patient remained consistently below 1 ng/mL. Only in April 2024, the PSA level rose again to 7.858. Subsequent diagnostic evaluations suggested that this abnormal PSA increase may have resulted from brain metastases of PC. Deceptively low PSA levels following ADT may lead to misdiagnosis, complicating subsequent PC diagnosis and treatment. Therefore, in patients with advanced PC and low PSA levels, postoperative follow-up and treatment should extend beyond the primary tumor site to assess potential metastases in other organs [29]. Clinically, more precise screening markers and strategies are essential for early detection and improved prognoses.

Brain metastasis is a complex process influenced by various risk factors. In lung cancer, brain metastases development has been linked to ADAM9 overexpression [30]. In breast cancer, ADAM8 overexpression and Ang-2 secretion by microvascular endothelial cells contribute to brain metastasis. ADAM8 facilitates endothelial cell migration by activating β1 integrin and promotes angiogenesis by inducing VEGF-A release and MMP-9 upregulation. Microvascular endothelial cells disrupt tight junctions by secreting Ang-2, thereby increasing the permeability of the blood-brain barrier and facilitating the tumor cell migration across it [31–33]. In response to these factors that may promote brain metastasis, various targeted therapies are being actively developed and tested in clinical trials. In PC, metastasis occurs through multiple paths. The most common route is direct extension from skull metastases, leading to subdural lesion formation [14,34]. Additionally, dissemination via the lymphatic system and blood circulation can result in subdural and intracranial lesions. Notably, metastasis through the paravertebral venous plexus allows tumor cells to bypass bones and other anatomical barriers [35]. However, no skull metastases were detected in this patient. In addition, brain metastases can profoundly affect the patient’s quality of life, due to complications such as headache, ataxia, visual field defects, cranial nerve palsy, paralysis, delirium, seizures, and coma [36]. Certain subtypes of PC, such as small-cell and neuroendocrine carcinomas, exhibit high malignancy and a strong propensity for brain metastasis. However, since adenocarcinoma is the predominant pathological type, brain metastases from PC are mostly adenocarcinoma ones in clinical practice [13,37]. Patients with intracranial metastases often have a poor prognosis, whether they have dura mater or brain parenchymal metastases [38]. A cohort study by Khani et al. reported that when intracranial metastasis of PC occurred, the median PSA was 50 ng/mL (range: 4.32–4308 ng/mL). The median time from PC diagnosis to the discovery of brain metastasis of PC was 56.6 months, and the median survival after the discovery of brain metastasis was only 11.2 months [39].

Conventional treatments for PC brain metastases (PCBM) include surgical resection, chemotherapy, and radiotherapy, administered alone or in combination [40]. For solitary brain metastases, the standard regimen consists of surgical resection followed by WBRT [9]. Selecting a specific treatment plan requires careful consideration of the clinical status and individual preferences of the patient. New-generation chemotherapy agents, such as docetaxel, have markedly improved symptoms and survival outcomes in PC; however, docetaxel has poor penetration across the blood-brain barrier and is unsuitable for intrathecal administration [35,41]. Conversely, the second-generation taxane cabazitaxel exhibits greater lipophilicity and lower affinity for the P-glycoprotein efflux pump, properties that enhance its ability to traverse the blood-brain barrier [7]. Studies have demonstrated that cabazitaxel combined with WBRT provides superior efficacy compared with either radiotherapy or docetaxel alone in treating PCBM, with a reported median progression-free survival of 11 months [42]. Given the neurotoxicity risks and neurocognitive decline associated with WBRT, stereotactic radiosurgery is increasingly utilized in clinical practice [27]. However, the therapeutic efficacy of external beam radiotherapy remains limited. Vickers et al. reported that patients receiving radiotherapy for brain or leptomeningeal metastases generally had an overall survival of <10 months, reflecting persistently poor outcomes [28]. In the present case, the patient underwent ADT and WBRT following surgical resection of brain metastases. The disease remained stable, with good tolerance and no significant adverse effects. PSA levels have consistently remained low (most recently 0.038 ng/mL); consequently, systemic chemotherapy with cabazitaxel was not pursued.

The precise mechanisms underlying PC brain metastasis remain unclear. Intracranial metastases frequently harbor high-frequency driver gene alterations, including TP53, PTEN, AR, RB1, and BRCA2, commonly shared with metastases at other anatomical sites [16,43]. This overlap suggests that therapeutic strategies guided by molecular and phenotypic profiling may be feasible, with PARP inhibitors (PARPi) and targeted radioligand therapy promising. Notably, PCBM displays a high prevalence of homologous recombination deficiency signatures, closely resembling those observed in PARPi-sensitive metastatic (mCRPC) [43]. Mechanistically, PARPi are likely to be effective in a subset of PCBM patients, particularly those with biallelic inactivation of BRCA1/2. However, prospective clinical evidence directly confirming this benefit is lacking. To address this gap, future clinical trials should incorporate patients with PCBM to evaluate their response to PARPi. Additionally, a cohort study by Gallon et al. demonstrated that PCBM is characterized by globally elevated CpG island methylation. Epigenetic profiles linked to driver gene alterations persist during brain metastases progression, providing new insights into systemic therapeutic strategies for mCRPC, including cases with intracranial involvement. In this case, the patient maintained long-term PSA stability during prior ADT and subsequently presented with multiple brain metastases. He underwent surgical resection followed by WBRT combined with continued ADT [15].

In summary, brain metastasis from PC is infrequent, and existing therapeutic strategies often result in suboptimal prognoses. Early diagnosis and timely intervention are crucial for improving prognosis and prolonging survival. Since PSA and tumor Gleason scores have limited value in predicting the future risk of disease progression, there is an urgent need for a detection index with high sensitivity, strong specificity, and lower overtreatment risk to detect PC. Furthermore, brain metastases generally arise in the setting of widespread systemic dissemination of PC [27]. In clinical practice, distinguishing between primary brain tumors and solitary brain metastases from PC can be challenging when only a single lesion is detected [13]. Accurate radiographic evaluation is essential for diagnosis. CT and MRI are widely used for brain tumor screening in clinical practice. However, brain imaging is not routinely incorporated into the standard PC screening. Consequently, in the early stages of metastasis, before the onset of neurological symptoms, brain metastases are often clinically overlooked. When the intracranial metastasis grows large enough to cause neurological symptoms, the prognostic outcomes are often unfavorable if brain metastases are diagnosed through brain imaging. Therefore, screening for brain metastases in patients with PC is clinically essential, particularly in those who have undergone endocrine therapy.

This case underscores the critical need for developing standardized clinical guidelines aimed at the early identification of atypical metastases in patients with high-risk PC. Although routine neuroimaging for all patients is not warranted, a low threshold for central nervous system investigation should be maintained in patients with advanced disease, particularly those with high-grade (Gleason score ≥ 8), high-volume bone disease, or treatment-resistant PSA progression.

This case report offers important clinical insights but has some limitations. First, the initial staging relied only on bone scintigraphy and pelvic MRI, without advanced whole-body imaging, such as PSMA-PET/CT, to exclude occult metastases. Second, the abrupt PSA increase to 7.858 ng/mL after nearly four years of stable suppression suggests that closer surveillance might have enabled earlier detection of biochemical recurrence before neurological symptoms emerged. Third, the absence of molecular characterization of the primary tumor and brain metastases limits understanding of the genomic drivers underlying this aggressive phenotype. These limitations highlight the challenges in managing high-risk patients and underscore the need for improved staging, rigorous monitoring, and molecular profiling in such cases. In the future, spatial transcriptomics and sequencing technologies will be employed to delineate the molecular and biological differences between brain metastases and their corresponding primary tumors. Future studies will employ spatial transcriptomics and sequencing to explore molecular differences between brain metastases and primary tumors.

## Supplementary Materials



## Data Availability

The datasets generated during and/or analyzed during the current study are available from the corresponding authors on reasonable request.

## Supplementary Materials

The supplementary material is available online at https://www.techscience.com/doi/10.32604/or.2025.066478/s1.
